# Ainda Procurando Entender o Papel do Ácido Úrico em Doenças Cardiovasculares

**DOI:** 10.36660/abc.20210390

**Published:** 2021-06-08

**Authors:** Gilson Soares Feitosa

**Affiliations:** 1 Escola Bahiana de Medicina SalvadorBA Brazil Escola Bahiana de Medicina , Salvador , BA - Brazil; Hospital Santa Izabel Santa Casa da Bahia SalvadorBA Brazil Hospital Santa Izabel da Santa Casa da Bahia , Salvador , BA - Brazil

**Keywords:** Doenças Cardiovasculares, Ácido Úrico, Hiperuricemia, Estresse Oxidativo, Obesidade, Endotélio, Fatores de Risco

Neste número dos Arquivos Brasileiros de Cardiologia, Zhu et al. ^1^ relatam, em estudo transversal, em uma população do norte da China, uma associação entre o nível sérico de ácido úrico e a presença de pré-hipertensão e hipertensão arterial, trazendo à tona, mais uma vez, um possível papel a ser melhor conhecido do ácido úrico na determinação de doenças cardiovasculares. No caso do presente estudo, chama a atenção que essa associação foi vista mesmo que com taxas bem reduzidas para os padrões de valores normais reconhecidos no Ocidente, que são ≥7,0mg/dL (para homens) e ≥6,3mg/dL (para mulheres), ^2^ enquanto os valores de referência como taxas elevadas no presente estudo foram de ≥4,75mg/dL (para homens) e ≥4,04mg/dL (para mulheres).

No Rio de Janeiro, em trabalhadores de ambos os sexos e com faixa etária predominante entre 50 e 59 anos, da Companhia de Geração e Distribuição de Energia no Rio de Janeiro, ^3^ a taxa média observada de ácido úrico foi de 4,7 ± 1,3mg/dL. ^4^

Já no estudo transversal PROCARDIO-UFV, também no Brasil, as médias foram de 4,4 ± 1,6mg/dL e de 5,4 ± 1,4mg/dL, quer se tratasse de indivíduo de baixo ou intermediário risco de Framingham, respectivamente. ^5^

Estudos dessa natureza realizados na Ásia demonstram valores mais reduzidos de apresentação da taxa de ácido úrico, seja por questões alimentares ou mesmo de origem genética. ^6 - 8^

O fato é que níveis séricos de AU (mg/dL) no grupo pré-hipertensão (3,5±1,1) e hipertensão (3,4±1,1) em comparação com o grupo controle (3,2±1,0) foram significativamente maiores no grupo de pré-hipertensos e hipertensos, a despeito do ajuste feito para fatores como idade, sexo, IMC, glicemia, e taxas lipídicas.

Uma questão que se levanta é se, diante do minúsculo grau de diferença entre os valores, concorreria tal achado para possíveis conjecturas de disfunção endotelial, como causa de doença cardiovascular nesses indivíduos. ^9 - 11^

Além disso, há potencial benefício do conhecimento para alguma ação terapêutica a ser oferecida.
