# Supplementary material for: Ainda Procurando Entender o Papel do Ácido Úrico em Doenças Cardiovasculares
Source: Arq Bras Cardiol. 2021 Jun 8;116(6):1079. [Article in Portuguese] doi: 10.36660/abc.20210390 (PMC8288542; doi:10.36660/abc.20210390)
Supplement: Translation

# Translation: Still Trying to Understand the Role of Uric Acid in Cardiovascular Diseases

**Authors:** Gilson Soares Feitosa

**Affiliations:** 1 Brazil Escola Bahiana de Medicina, Salvador, BA - Brazil; Brazil Hospital Santa Izabel da Santa Casa da Bahia, Salvador, BA - Brazil

**Keywords:** Cardiovascular Diseases, Uric Acid, Hyperurisemia, Oxidative Stress, Obesity, Endothelium, Risk Factors

In this issue of *Arquivos Brasileiros de Cardiologia* , Zhu et al.^1^ report an association between uric acid and the presence of pre-hypertension or hypertension in a cross-sectional study done in a population in North China, bringing about, once more, a possible role of uric acid in the determination of cardiovascular disease. In the study, the authors call our attention to the possibility of a relationship even though the measured uric acid serum level is relatively low as compared to what is referred to as normal in several Western populations, which presents a cutoff value of ≥7.0 mg/dL for men and ≥6.3 mg/dL for women^2^ in the United States and also so considered, a cutoff value of ≥7.0 mg/dL for men and ≥ 6.0 mg/dL for women in a Brazilian population of healthy individuals aged 20 to 55, in Rio de Janeiro,^3^ whereas in their the cut off values were ≥4.75 mg/dL and ≥4.04 mg/dL for men and women, respectively.

In workers from the Company of Generation and Distribution of Energy in Rio de Janeiro, Brazil, from both sexes, aged predominantly between 50 and 59, the mean uric acid level was 4.7±1.3 mg/dL.^4^

In another study in Brazil, in a cross-sectional study named PROCARDIO-UFV, the mean serum uric acid levels were 4.4±1.6 mg/dL and 5.4±1.4 mg/dL in low and intermediate Framingham risk score, respectively.^5^

Similar studies that come from Asia display lesser uric acid values in the general population, and eating habits or genetic factors are speculated as its cause.^6 - 8^

In the study the values of 3.5±1.1 mg/dL in pre-hypertensives and 3.4±1.1 mg/dL in hypertensives were significantly higher than 3.2±1.0 mg/dL in the control group, regardless of the adjustments made for confounding factors such as age, sex, body mass index, glucose, and lipid levels.

A question that arises is whether such a minuscule difference of values would justify the conjectured changes in endothelial function as a cause of cardiovascular disease in such individuals,^9 - 11^ in addition to its potential therapeutic target.
